# Astragaloside IV from *Astragalus membranaceus* Fisch. ex Bunge Mitigates DSS-Induced Colitis via Anti-Inflammatory and Antioxidant Modulation of the Gut–Liver–Brain Axis

**DOI:** 10.3390/antiox15040474

**Published:** 2026-04-10

**Authors:** Woo-Gyun Choi, Byung Joo Kim

**Affiliations:** Department of Longevity and Biofunctional Medicine, Pusan National University School of Korean Medicine, Yangsan 50612, Republic of Korea; ak0510@pusan.ac.kr

**Keywords:** chronic intestinal inflammation, DSS-induced colitis, Astragaloside IV, NF-κB/MAPK signaling cascade, inflammatory modulation, oxidative damage protection

## Abstract

Background: *Astragalus membranaceus* Fisch. ex Bunge has long been used in East Asian medicine for gastrointestinal disorders and immune modulation. Astragaloside IV (AS-IV), a major bioactive saponin from its roots, exhibits potent anti-inflammatory and antioxidant activities, yet its protective effects against inflammatory bowel disease (IBD)-associated multi-organ damage via the gut–liver–brain axis remain unclear. Methods: Experimental colitis was induced in C57BL/6N mice by administering 5% dextran sulfate sodium (DSS) in drinking water for seven days. AS-IV (100 mg/kg/day) was orally administered during DSS exposure. Disease severity was evaluated using body weight, colon length, disease activity index, and histopathology. Inflammatory cytokines and oxidative stress markers were measured using ELISA, and NF-κB and MAPK signaling were analyzed through Western blotting and immunohistochemistry in colonic, hepatic, and brain tissues. Results: AS-IV significantly alleviated DSS-induced weight loss, disease activity, and colon shortening, while improving intestinal histopathological damage. AS-IV also reduced systemic pro-inflammatory cytokine levels and oxidative stress. Mechanistically, AS-IV was associated with a reduced expression of phosphorylated NF-κB and MAPK proteins, including p-NF-κB, p-IκBα, p-ERK, p-JNK, and p-p38, across the colon, liver, and brain. Conclusions: AS-IV attenuates DSS-induced multi-organ inflammation via gut–liver–brain axis modulation through NF-κB and MAPK pathway inhibition in experimental colitis models.

## 1. Introduction

Astragaloside IV (AS-IV) is a major bioactive saponin isolated from the roots of *Astragalus membranaceus* Fisch. ex Bunge, a medicinal plant extensively utilized in traditional East Asian medicine. Over the past decade, accumulating evidence has demonstrated that AS-IV exerts diverse pharmacological activities, including antioxidative, anti-inflammatory, neuroprotective, antifibrotic, and antineoplastic effects. Notably, AS-IV effectively attenuates oxidative stress by suppressing excessive reactive oxygen species (ROS) production while concomitantly modulating inflammatory mediator release, positioning it as a promising therapeutic candidate for oxidative stress-related inflammatory diseases [1]. *Astragalus membranaceus* Fisch. ex Bunge, known in Traditional Chinese Medicine as Radix Astragali (Huangqi), is derived from the dried roots of leguminous plants belonging to the *Fabaceae* family [2]. Historical botanical records indicate that this herb was first documented in the Shennong’s Classic of Materia Medica during the Han Dynasty (approximately 200–300 BCE) and has been continuously used for more than two millennia as a tonic herb to enhance vitality and resistance against disease [3].

Inflammatory bowel disease (IBD) is an umbrella term for chronic inflammatory gastrointestinal conditions, most notably Crohn’s disease (CD) and ulcerative colitis (UC), which together represent a major contributor to morbidity worldwide [4]. Patients typically present with recurring abdominal pain, diarrhea, rectal bleeding, and intestinal hemorrhage, with disease activity fluctuating between periods of flare and remission [5]. Although the etiology of IBD is multifactorial, increasing evidence highlights oxidative stress as a critical pathogenic factor driving mucosal injury, immune dysregulation, and disease progression [6].

An excessive generation of ROS disrupts intestinal epithelial integrity and activates redox-sensitive inflammatory signaling pathways, particularly nuclear factor kappa B (NF-κB) and mitogen-activated protein kinase (MAPK) cascades [7,8]. Activation of these pathways leads to the enhanced transcription of genes encoding pro-inflammatory mediators, including tumor necrosis factor-α (TNF-α), cyclooxygenase-2 (COX-2), interleukins (IL-1β and IL-6), and inducible nitric oxide synthase (iNOS) [9,10]. Moreover, MAPK signaling via extracellular signal-regulated kinase (ERK) 1/2, p38, and c-Jun N-terminal kinase (JNK) further amplifies inflammatory responses under oxidative stress conditions [11]. Persistent redox imbalance and inflammatory amplification ultimately result in epithelial barrier dysfunction and chronic intestinal inflammation characteristic of IBD [12,13,14]. Conventional treatments for IBD, including aminosalicylates and corticosteroids, focus largely on controlling inflammation and relieving symptoms. Nevertheless, their use is frequently associated with considerable side effects and an insufficient durability of response [15,16,17], highlighting the pressing demand for safer therapeutic alternatives that address upstream inflammatory and oxidative pathways.

Given its potent antioxidative and anti-inflammatory properties, AS-IV represents a compelling candidate for IBD therapy. Nevertheless, systematic investigations delineating its protective effects against oxidative stress-driven intestinal inflammation and its underlying molecular mechanisms in dextran sulfate sodium (DSS)-induced colitis models remain insufficient. Accordingly, this study was undertaken to assess the efficacy of AS-IV in an experimental colitis setting, with a focus on its ability to regulate oxidative stress and NF-κB/MAPK-mediated inflammatory signaling throughout the gut–liver–brain axis [18,19].

## 2. Materials and Methods

### 2.1. Experimental Animals and Ethical Approval

C57BL/6N mice (38 male, 20–22 g) were obtained from OrientBio Corporation (Seongnam, Republic of Korea). No additional environmental enrichment was provided during the experimental period. Standard SPF housing conditions were maintained for all animals, including an ambient temperature of 22 ± 2 °C, a relative humidity range of 50–60%, and a 12 h light/dark cycle. Animals were provided with Purina Rodent Chow (Purina Korea Inc., Product No. 38057, Pyengtaek, Republic of Korea) and water ad libitum throughout the study. No genetic modifications were present; all animals were wild-type. Mice had no prior procedures before the start of this study. Astragaloside IV (AS-IV; purity >98.0%, HPLC grade) was purchased from Tokyo Chemical Industry (Tokyo, Japan). After a 7-day acclimatization period, mice were randomly assigned to the following experimental groups (*n* = 6–7 per group): (1) vehicle control group receiving normal drinking water (*n* = 7); (2) DSS group receiving 5% DSS in drinking water (*n* = 7); and (3–6) DSS + AS-IV treatment groups receiving 5% DSS with oral administration of AS-IV at doses of 10 (*n* = 6), 50 (*n* = 6), 100 (*n* = 6), or 200 mg/kg (*n* = 6), respectively. Based on the dose–response results, AS-IV at 100 mg/kg was selected for all subsequent analyses beyond the dose–response assessment. The primary outcome measure was the disease activity index (DAI), which reflects colitis severity and was used to determine the sample size for the study. Experimental colitis was induced by administering 5% (*w*/*v*) DSS in drinking water for 7 consecutive days. AS-IV was freshly prepared prior to each administration and delivered once daily by oral gavage, with dosages adjusted according to individual body weight, following previously established protocols [20]. Throughout the experimental period, body weight, food intake, and fecal characteristics were monitored daily. Disease progression and general health status were evaluated based on changes in body weight, stool consistency, and the presence of fecal blood [21]. At the end of the experimental period, mice were euthanized, and biological samples including whole blood, liver tissue, intestinal segments, and brain tissues were collected for subsequent analyses. All animals were housed under identical conditions, and treatments and measurements were conducted at similar times of the day. Investigators were aware of group allocation during the conduct of the experiment. However, outcome assessments and data analyses were performed in a blinded manner. No expected or unexpected adverse events were observed in any experimental group. Humane endpoints were established prior to the experiment. Mice were monitored daily for body weight loss, diarrhea, rectal bleeding, and general signs of distress. Any animal showing more than 25% body weight loss or severe clinical symptoms was euthanized immediately. When all experimental procedures were complete, animals were humanely killed by gradual-fill CO_2_ inhalation in accordance with institutional guidelines.

### 2.2. Assessment of Disease Severity and Activity Index

Daily assessments included measurement of body weight (g), food intake (mL), and evaluation of stool consistency throughout the treatment period. Colonic tissue damage was graded using a standardized scoring system ranging from 0 to 4 based on macroscopic pathological features, according to previously established criteria [22]. The disease activity index (DAI) was calculated by integrating multiple clinical parameters, as described in Supplementary Materials Table S1.

### 2.3. Histological Processing and Plasma Alanine Aminotransferase Quantification

Tissue samples from the colon, liver, and brain were immersion-fixed in 10% neutral-buffered formalin and subsequently subjected to routine histopathological processing [22]. Paraffin-embedded sections were prepared and stained with H&E for microscopic evaluation. Histological evaluation was performed using a polarized light microscope (Leica Microsystems, Bensheim, Germany). Quantitative image analysis was conducted using a commercial colorimetric detection system (Tio Vision, Milpitas, CA, USA) in accordance with the manufacturer’s instructions [23].

### 2.4. Measurement of Circulating Endotoxin, Lipid Metabolites, and Oxidative Biomarkers

Plasma lipopolysaccharide (LPS) levels were quantified using a Pierce™ Chromogenic Endotoxin Quantitation Kit (Thermo Fisher Scientific, Waltham, MA, USA, Cat. 88282) according to the manufacturer’s instructions. Hepatic triglyceride content was measured using a commercial assay kit (Asan Co., Ltd., Gimpo, Republic of Korea, Cat. AM157S). Plasma ROS levels were assessed using 2′,7′-dichlorofluorescein diacetate (DCF-DA, Thermo Fisher Scientific, Waltham, MA, USA, Cat. D399) following established fluorometric protocols [24,25].

### 2.5. Inflammatory Cytokine Quantification Methodology

Plasma concentrations of IL-1β and TNF-α were quantified using commercial sandwich ELISA kits (R&D Systems, Minneapolis, MN, USA; IL-1β, Cat. DY201; TNF-α, Cat. DTA00D) according to the manufacturer’s instructions. Nitric oxide metabolite (nitrite/nitrate) levels were determined using a colorimetric assay kit (Cayman Chemical, Ann Arbor, MI, USA, Cat. 780001) following established protocols.

### 2.6. Immunoblot Analysis

Hepatic, colonic, and neural tissues were collected from each mouse and individually homogenized in ice-cold RIPA lysis buffer. Protein concentrations were determined for each sample, and equal amounts of protein from individual mice within the same group were combined to prepare pooled lysates (50 μg total protein per group). Each pooled lysate was analyzed in triplicate (three technical replicates), and data are expressed as mean ± SEM of the three replicates. The pooled proteins were then separated through SDS-PAGE and transferred onto nitrocellulose membranes (Bio-Rad, Hercules, CA, USA). Membranes were blocked with 5% skim milk in TBST (0.05% Tween-20) for 30 min at room temperature, and then incubated with primary antibodies overnight at 4 °C. After washing with TBST five times for 5 min each, membranes were incubated with appropriate secondary antibodies for 1 h at room temperature, followed by another five washes with TBST (5 min each). Protein bands were visualized using an enhanced chemiluminescence system (Thermo Fisher Scientific, Waltham, MA, USA) and quantified through densitometry using Fusion Solo software (V18.02, Vilber, Paris, France). Relative protein expression was normalized to GAPDH. Primary antibodies and dilutions were as follows: 3-nitrotyrosine (1:3000, Abcam, Cambridge, UK, ab61392), Bax (1:1000, Santa Cruz, Santa Cruz, CA, USA, SC-7480), β-catenin (1:1000, Santa Cruz, SC-7963), cleaved caspase-3 (1:1000, Cell Signaling, Danvers, MA, USA, #9661), COX-2 (1:1000, Santa Cruz, SC-376861), CYP2E1 (1:3000, Abcam, ab28146), E-cadherin (1:1000, Santa Cruz, SC-8426), GAPDH (1:1000, Santa Cruz, SC-47724), γ-catenin (1:1000, Santa Cruz, SC-59986), iNOS (1:3000, Abcam, ab136918), occludin (1:1000, Santa Cruz, SC-271842), p-ERK (1:1000, Santa Cruz, SC-7383), p-IκB-α (1:1000, Santa Cruz, SC-8404), p-JNK (1:1000, Santa Cruz, SC-6254), p-NF-κB (1:1000, Santa Cruz, SC-271908), p-p38 (1:1000, Santa Cruz, SC-7973), and ZO-1 (1:3000, Abcam, ab216880).

### 2.7. Programmed Cell Death Assessment

Hepatic tissues were paraffin-embedded, sectioned, and subjected to TUNEL (terminal deoxynucleotidyl transferase dUTP nick-end labeling) staining using the ApopTag^®^ Peroxidase In Situ Apoptosis Detection Kit (Sigma-Aldrich, Ann Arbor, MI, USA) according to the manufacturer’s instructions [26].

### 2.8. Microbial 16S Ribosomal RNA Sequence Analysis and Bioinformatics

Cecal contents from individual mice were rapidly frozen at −80 °C. Bacterial DNA was extracted using the Mag-Bind^®^ Universal Pathogen DNA Kit (MACROGEN, Seoul, Republic of Korea). Microbial 16S rRNA gene sequencing and subsequent bioinformatic analyses were performed by MACROGEN Inc. (http://www.macrogen.com) [27]. Raw sequencing data have been deposited in the NCBI SRA under BioProject accession number PRJNA1426157.

### 2.9. Cellular Culture and Immunofluorescence Microscopy

N2a neuroblastoma cells (CCL-131, ATCC, Manassas, VA, USA), T84 human intestinal epithelial cells, and AML12 murine hepatocytes were cultured in Dulbecco’s Modified Eagle Medium (DMEM) supplemented with 10% heat-inactivated fetal bovine serum (FBS) and 1% penicillin–streptomycin. For tight junction protein visualization, T84 cells were stained with anti-ZO-1 primary antibody (1:200, Abcam, ab216880) followed by Alexa Fluor 488-conjugated anti-rabbit secondary antibody (Thermo Fisher Scientific, Waltham, MA, USA, Cat. A11008), and with anti-occludin primary antibody (1:200, Santa Cruz, SC-271842) followed by Alexa Fluor 488-conjugated anti-mouse secondary antibody (Thermo Fisher Scientific, Waltham, MA, USA, Cat. A11001). For CYP2E1 detection, AML12 cells were incubated overnight at 4 °C with anti-CYP2E1 primary antibody (1:200, Abcam, ab28146), followed by Alexa Fluor 488-conjugated anti-rabbit secondary antibody (Thermo Fisher Scientific, Waltham, MA, USA, Cat. A11008). Nuclei were counterstained with 1 μg/mL DAPI for 5 min, and apoptosis was assessed by co-staining with anti-cleaved caspase-3 primary antibody (1:200, Cell Signaling, #9661) followed by Alexa Fluor 488-conjugated anti-rabbit secondary antibody (Thermo Fisher Scientific, Waltham, MA, USA, Cat. A11008) and DCF-DA. After washing and mounting with VectaShield^®^ fluorescence mounting medium (Vector Laboratories, Burlingame, CA, USA), images were captured using a confocal microscope.

### 2.10. Statistical Analysis

All data are expressed as mean ± standard error of the mean (SEM). Group comparisons were carried out using one-way ANOVA with Dunnett’s post hoc test in SPSS version 26.0, and differences were considered statistically significant at *p* < 0.05.

## 3. Results

### 3.1. AS-IV Attenuates Body Weight Loss and Preserves Colon Length in DSS-Induced Acute Colitis

**Figure 1:**
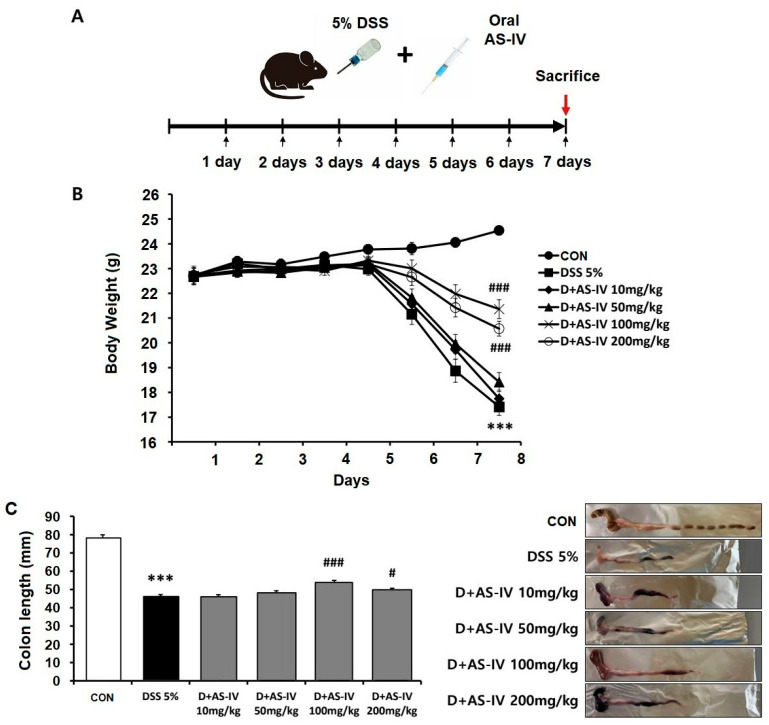
Dose–response evaluation of AS-IV effects on body weight and colon length in DSS-induced acute colitis model. (**A**) Experimental timeline of 5% DSS administration and daily AS-IV gavage (10, 50, 100, 200 mg/kg, *n* = 6) for 7 days. (**B**) Body weight recovery was significantly improved by 100 and 200 mg/kg AS-IV, with 100 mg/kg showing optimal efficacy. (**C**) Colon length restoration and representative images indicate 100 mg/kg AS-IV provided maximal benefit. Data are presented as means ± SEM. *** *p* < 0.005 vs. CON group; # *p* < 0.05, ### *p* < 0.005 vs. DSS group.

To evaluate the anti-inflammatory and antioxidative effects of AS-IV across a range of previously reported experimental doses (10–200 mg/kg) [1,28], experimental colitis was induced by administering 5% DSS in drinking water for seven days (Figure 1A). The therapeutic potential of AS-IV was assessed in this DSS-induced acute colitis model by monitoring body weight changes and colon length across different treatment concentrations. Analysis of body weight trajectories (Figure 1B) revealed that AS-IV administration at 100 mg/kg and 200 mg/kg significantly promoted recovery following DSS challenge, with the 100 mg/kg dose exhibiting superior therapeutic outcomes. Notably, the 200 mg/kg dose showed significantly less efficacy than the 100 mg/kg dose, indicating that the optimal therapeutic effect was achieved at 100 mg/kg. Examination of colon length (Figure 1C) showed marked shortening in DSS-exposed mice (46.17 ± 1.01 mm; *** *p* < 0.005), indicative of acute colitis. Treatment with 100 mg/kg AS-IV significantly improved colon length compared to the DSS group (53.83 ± 1.11 mm; ### *p* < 0.005), whereas the 200 mg/kg dose produced only modest improvement (49.83 ± 0.79 mm; # *p* < 0.05). These results identify 100 mg/kg as the optimal dose for achieving maximal therapeutic efficacy while minimizing unnecessary drug exposure. Accordingly, AS-IV at 100 mg/kg was selected for all subsequent experiments.

### 3.2. AS-IV Ameliorates Clinical Manifestations, Inflammatory Status, and Oxidative Stress in DSS-Challenged Acute Colitis Mice

**Figure 2:**
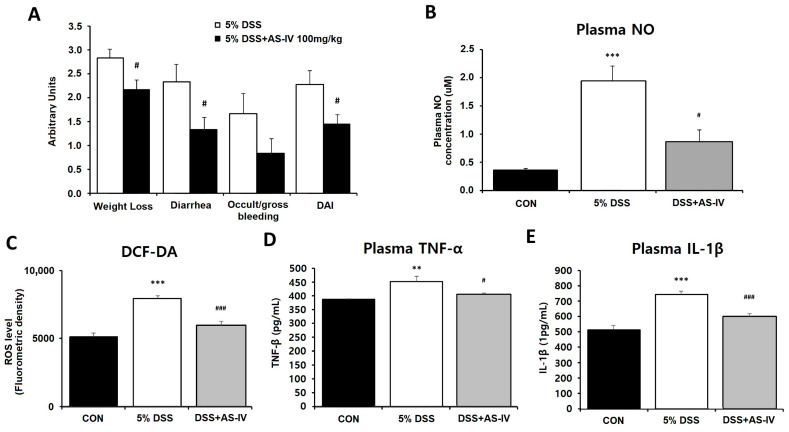
AS-IV alleviates clinical symptoms, inflammation, and oxidative stress in DSS-induced colitis. (**A**) DAI, including body weight loss, diarrhea, and bleeding, in CON, DSS, and DSS + AS-IV (100 mg/kg) groups (*n* = 6). The CON group is not shown in the graph as all DAI scores were 0 throughout the experimental period. (**B**) Plasma nitric oxide (NO) levels. (**C**) Colonic ROS levels assessed using DCF-DA fluorescence. (**D**) Plasma TNF-α concentrations. (**E**) Plasma IL-1β concentrations. Data are presented as means ± SEM. ** *p* < 0.01, *** *p* < 0.005 vs. CON group; # *p* < 0.05, ### *p* < 0.005 vs. DSS group. Statistical comparison between CON and DSS + AS-IV groups was not performed for NO, ROS, and IL-1β levels, as these did not return to control-like levels following AS-IV treatment.

Observable clinical deterioration, including body weight loss, loose stools, occult intestinal bleeding, and elevated DAI scores, was systematically recorded throughout the observation period to evaluate the therapeutic potential of AS-IV in DSS-induced acute colitis. Animals treated with DSS plus AS-IV exhibited moderate reductions in DAI values compared to DSS-only mice (Figure 2A). Oxidative stress parameters in colonic tissue were assessed by quantifying nitric oxide (NO) and ROS levels, alongside pro-inflammatory cytokines. DSS exposure significantly increased plasma TNF-α and IL-1β levels, whereas co-administration of AS-IV markedly reduced NO and ROS levels compared to the DSS group (Figure 2B,C), restored TNF-α levels to control-like levels (Figure 2D), and significantly reduced IL-1β levels compared to the DSS group (Figure 2E). These results indicate that AS-IV exerts both anti-inflammatory and antioxidative effects in mice with DSS-induced colitis.

### 3.3. AS-IV Suppresses Lipopolysaccharide Concentrations and Restores Bacterial Community Structure in DSS-Challenged Acute Colitis Mice

**Figure 3:**
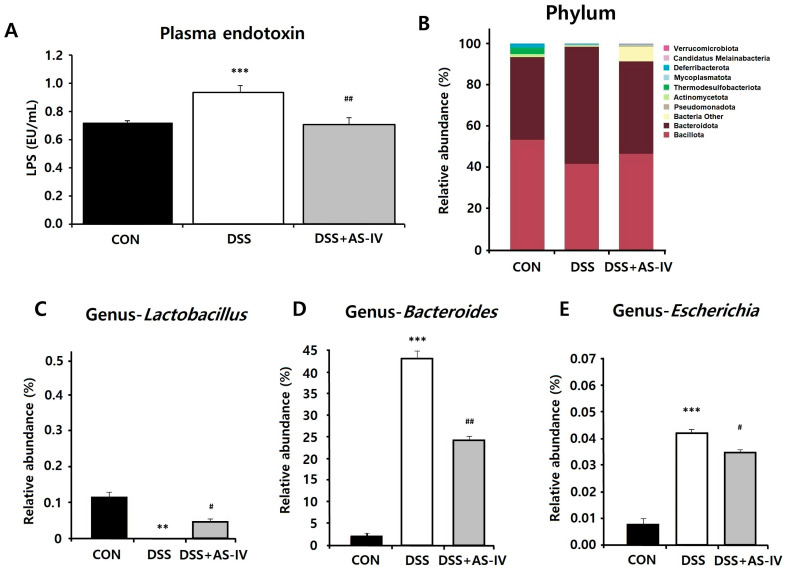

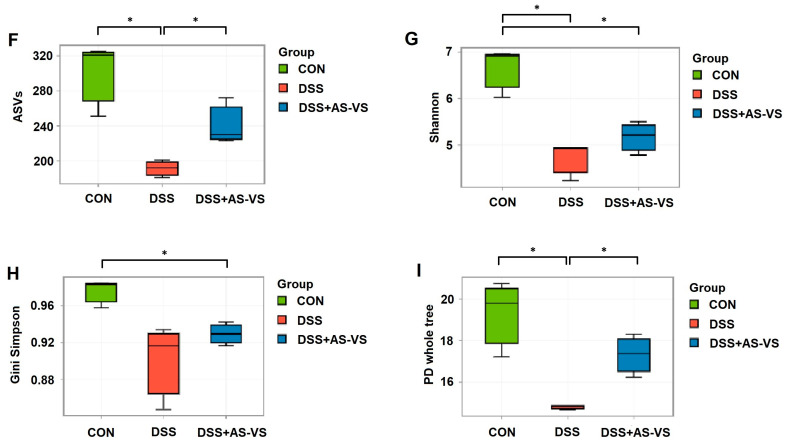
AS-IV reduces plasma endotoxin levels and modulates gut microbiota in DSS-induced colitis. (**A**) Plasma LPS concentrations in CON, DSS, and DSS + AS-IV (100 mg/kg) groups (*n* = 6). (**B**) Phylum-level bacterial composition showing relative abundance of major phyla. Genus-level relative abundance of *Lactobacillus* (**C**), *Bacteroides* (**D**), and *Escherichia* (**E**) (*n* = 6). (**F**–**I**) Alpha diversity indices including ASVs (**F**), Shannon index (**G**), Gini–Simpson (**H**), and PD whole tree (**I**) across experimental groups. Significant differences between groups are indicated by brackets with asterisks. Data are presented as means ± SEM. ** *p* < 0.01, *** *p* < 0.005 vs. CON group; # *p* < 0.05, ## *p* < 0.01 vs. DSS group. * *p* < 0.05 between indicated groups (FDR-corrected).

LPS, a component of Gram-negative bacterial cell walls, contributes to elevated systemic concentrations during DSS-induced colitis, compromising epithelial barrier integrity. DSS exposure also disrupts the intestinal epithelial layer and promotes microbial translocation into the systemic circulation [29]. In our study, plasma endotoxin levels were significantly increased in DSS-challenged mice compared to controls, whereas co-administration of AS-IV substantially reduced endotoxin concentrations (Figure 3A). Cecal bacterial populations were analyzed to assess microbial diversity across experimental groups. DSS exposure resulted in increased *Bacteroidota* abundance and decreased *Bacillota* populations relative to controls (Figure 3B). *Lactobacillus* sp. was significantly depleted following DSS treatment but partially increased compared to the DSS group following AS-IV treatment, suggesting a partial shift toward baseline microbial composition (Figure 3C,D). Additionally, DSS-challenged mice exhibited elevated *Escherichia coli* populations, which were markedly reduced by AS-IV intervention (Figure 3E). These findings suggest that alterations in microbial community composition play a critical role in maintaining intestinal ecosystem balance during colitis [30]. Specifically, AS-IV treatment appears to promote beneficial bacterial populations while suppressing pathogenic species, thereby supporting intestinal barrier integrity and overall gut homeostasis. To further characterize the effect of AS-IV on gut microbial diversity, alpha diversity was assessed using four indices: ASVs, Shannon index, Gini–Simpson, and PD whole tree (Figure 3F–I). DSS-challenged mice exhibited significantly reduced alpha diversity compared to controls across multiple indices, including ASVs (FDR-adjusted *p* = 0.034), Shannon index (FDR-adjusted *p* = 0.034), and PD whole tree (FDR-adjusted *p* = 0.034), indicating a marked reduction in microbial richness and evenness following DSS exposure. AS-IV treatment partially restored microbial diversity, with significant recovery observed in the ASV index (FDR-adjusted *p* = 0.034 vs. DSS) and PD whole tree (FDR-adjusted *p* = 0.034 vs. DSS). These findings suggest that AS-IV exerts a protective effect on gut microbial community structure during DSS-induced colitis.

### 3.4. AS-IV Preserves Colonic Architecture, Suppresses Oxidative Stress Indicators, and Inhibits MAPK Signaling in Colon Tissue from DSS-Induced Acute Colitis Mice

**Figure 4:**
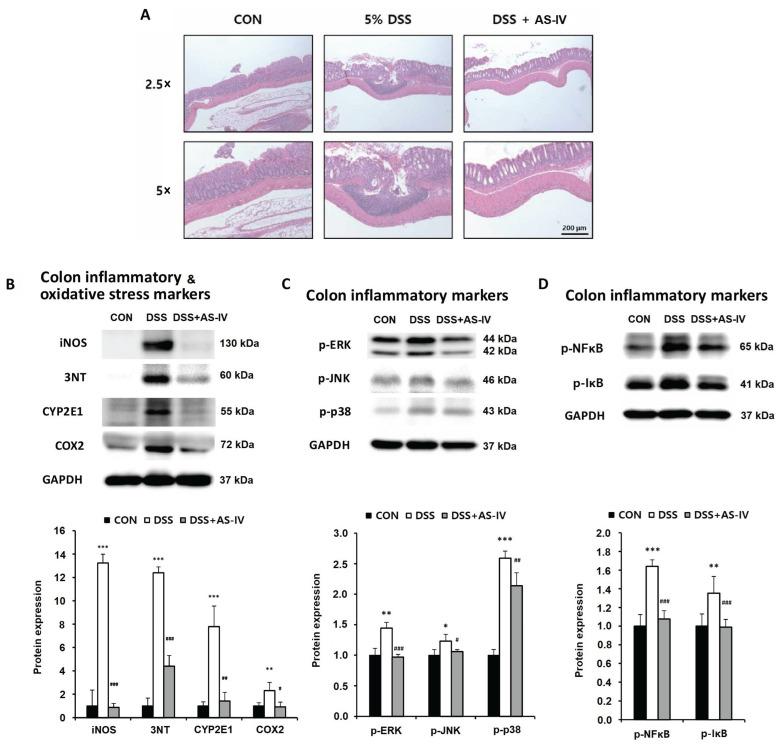
AS-IV preserves colon architecture and inhibits oxidative stress and inflammatory signaling in DSS-induced colitis. Colon tissue samples were obtained from CON, DSS, and DSS + AS-IV (100 mg/kg) groups. (**A**) Representative H&E-stained colon sections showing crypt structure and inflammatory infiltration (2.5× and 5×, scale bar = 200 μm, *n* = 6). (**B**) Western blot quantification of inflammatory and oxidative stress markers (iNOS, 3-nitrotyrosine, CYP2E1, COX-2) in colon tissue; GAPDH as loading control. (**C**) Western blot quantification of MAPK pathway proteins (p-ERK, p-JNK, p-p38). (**D**) Western blot quantification of NF-κB pathway proteins (p-NF-κB, p-IκBα). Data are presented as means ± SEM. * *p* < 0.05, ** *p* < 0.01, *** *p* < 0.005 vs. CON group; # *p* < 0.05, ## *p* < 0.01, ### *p* < 0.005 vs. DSS group (*n* = 3 technical replicates from pooled lysates in Western blot).

Microscopic examination of colonic tissues revealed pronounced architectural alterations in DSS-exposed mice, including crypt damage, mucosal inflammatory cell infiltration, and submucosal disruption. In contrast, mice treated with DSS plus AS-IV (100 mg/kg) exhibited preserved crypt architecture and reduced inflammatory cell infiltration in both mucosal and submucosal layers (Figure 4A). DSS administration significantly increased colonic protein expression of iNOS, 3-nitrotyrosine, CYP2E1, and COX-2, indicating enhanced inflammatory and oxidative/nitrosative stress responses. AS-IV treatment markedly reduced these inflammatory and oxidative stress markers in DSS-challenged mice (Figure 4B). Given the pivotal role of MAPK signaling in inflammatory responses [31], we assessed the levels of phosphorylated MAPK components and NF-κB-related proteins in colonic tissues. DSS exposure substantially elevated the levels of p-ERK, p-JNK, p-p38, p-NF-κB, and p-IκBα, whereas co-administration of AS-IV reduced the expression of these phosphorylated proteins, with a partial reduction observed for p-p38 (Figure 4C,D). These results demonstrate that AS-IV mitigates DSS-induced oxidative/nitrosative stress and inhibits activation of the NF-κB/MAPK inflammatory signaling pathways in colonic tissue.

### 3.5. AS-IV Restores Intestinal Tight Junction and Adherens Junction Protein Concentrations in DSS-Induced Acute Colitis Mice and Provides Protection Against LPS-Mediated Barrier Disruption In Vitro

**Figure 5:**
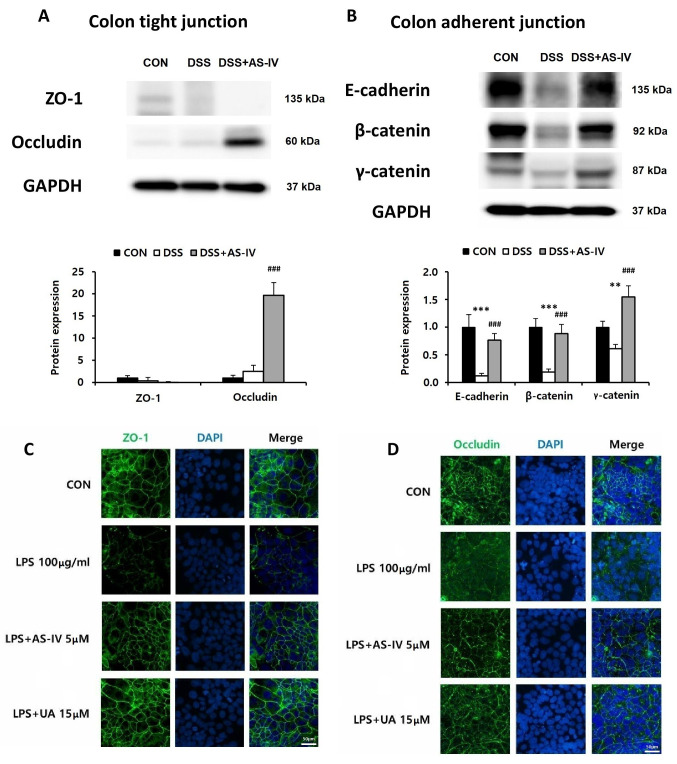
AS-IV restores intestinal junction proteins in DSS-induced colitis and protects against LPS-induced barrier disruption in vitro. Colon tissue samples were obtained from CON, DSS, and DSS + AS-IV (100 mg/kg) groups. (**A**) Western blot of tight junction proteins (ZO-1, occludin) in colon tissue; GAPDH as loading control (*n* = 3 technical replicates from pooled lysates). (**B**) Western blot of adherens junction proteins (E-cadherin, β-catenin, γ-catenin) in colon tissue; GAPDH as loading control (*n* = 3 technical replicates from pooled lysates). Representative immunofluorescence images of ZO-1 (**C**) and occludin (**D**) in T84 cells treated with CON, LPS (100 μg/mL), LPS + AS-IV (5 μM), or LPS + ursolic acid (UA, 15 μM); nuclei stained with DAPI (blue) (*n* = 6). Data are presented as means ± SEM. ** *p* < 0.01, *** *p* < 0.005 vs. CON group; ### *p* < 0.005 vs. DSS or LPS group.

IBD is associated with increased intestinal permeability and reduced expression of tight junction (TJ) proteins, as well as decreased levels of adherens junction (AJ) components in intestinal epithelial tissue [32]. To assess the effect of AS-IV (100 mg/kg), we measured TJ proteins (ZO-1 and occludin) and AJ components (E-cadherin, β-catenin, and γ-catenin) using Western blotting. DSS exposure did not significantly alter ZO-1 or occludin levels compared to the CON group (Figure 5A). However, AS-IV treatment significantly increased occludin levels compared to the DSS group, while ZO-1 levels remained unchanged across all groups (Figure 5A). AJ protein expression was markedly decreased by DSS exposure, which was substantially restored by AS-IV treatment (Figure 5B). These changes corresponded with enhanced intestinal permeability in DSS-challenged mice, whereas AS-IV mitigated both AJ protein reduction and barrier disruption. Intestinal-derived bacterial endotoxin (LPS) is a potent inflammatory compound capable of compromising epithelial barrier integrity, and previous studies suggest that AS-IV attenuates LPS-induced damage in T84 intestinal epithelial cells [33,34]. To test this, T84 cells were treated with vehicle, LPS, or LPS plus AS-IV (5 μM). Immunofluorescence analysis showed that LPS-induced reductions in ZO-1 and occludin expression were prevented by AS-IV co-treatment (Figure 5C,D). Although ZO-1 levels were not significantly altered across all groups in vivo, AS-IV significantly increased occludin levels compared to the DSS group and substantially improved AJ protein expression, suggesting that barrier protection is primarily reflected through AJ integrity maintenance under the acute DSS colitis conditions employed in this study. Furthermore, in vitro experiments using T84 cells demonstrated that AS-IV prevented LPS-induced reductions in ZO-1 and occludin expression, indirectly supporting the potential of AS-IV to preserve TJ protein integrity under inflammatory conditions. Taken together, these results indicate that AS-IV confers intestinal barrier protection through complementary AJ-mediated in vivo effects and TJ-preserving effects observed in vitro.

### 3.6. AS-IV Prevents Hepatic Dysfunction and Apoptotic Cell Death in DSS-Induced Acute IBD Mice

**Figure 6:**
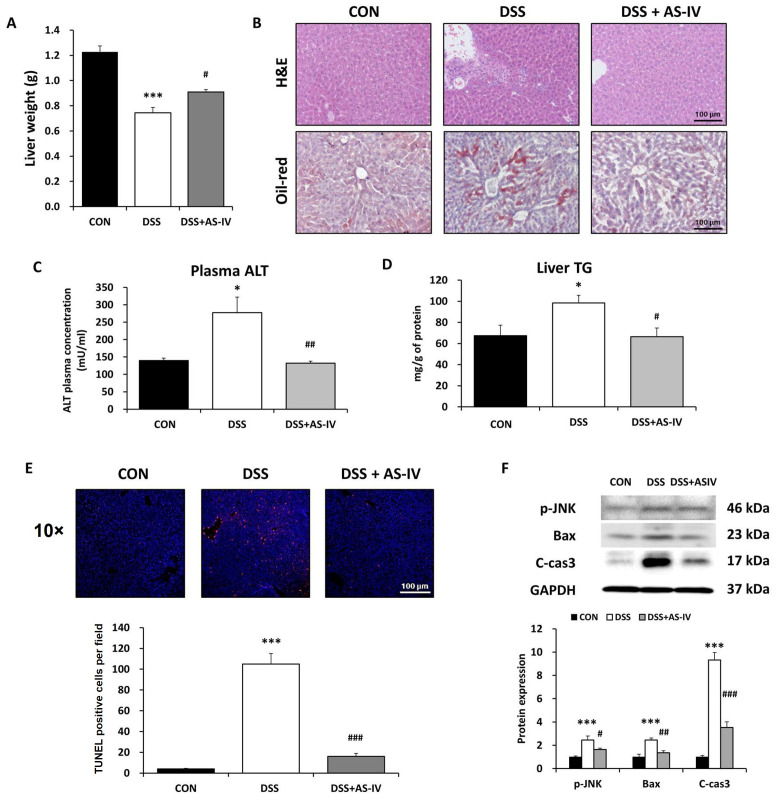
AS-IV prevents liver injury and apoptosis in DSS-induced colitis. (**A**) Liver weight in CON, DSS, and DSS + AS-IV (100 mg/kg) groups (*n* = 6). (**B**) Representative H&E (upper) and Oil-red O (lower)-stained liver sections showing architecture and lipid accumulation (scale bar = 100 μm, *n* = 6). (**C**) Plasma ALT activity. (**D**) Hepatic triglyceride (TG) content. (**E**) Representative TUNEL staining (10×) and quantification of apoptotic cells (scale bar = 100 μm, *n* = 6). (**F**) Western blot of apoptosis-related proteins (p-JNK, Bax, cleaved caspase-3, *n* = 3 technical replicates from pooled lysates); GAPDH as loading control. Data are presented as means ± SEM. * *p* < 0.05, *** *p* < 0.005 vs. CON group; # *p* < 0.05, ## *p* < 0.01, ### *p* < 0.005 vs. DSS group.

DSS-induced colitis elevates portal LPS concentrations, promoting hepatic inflammation and structural damage [35]. To investigate AS-IV’s protective effects on DSS-induced hepatic dysfunction, liver injury markers were assessed on day seven with or without AS-IV treatment. AS-IV partly prevented DSS-induced reductions in liver mass and necrotic tissue formation (Figure 6A,B). Circulating ALT levels were significantly higher in DSS-treated animals than in controls, and this elevation was markedly attenuated by AS-IV administration (Figure 6C). Hepatic triglyceride accumulation, assessed by Oil Red O staining, was increased in DSS-exposed mice and attenuated by AS-IV administration (Figure 6B,D). TUNEL staining revealed a significant increase in hepatocyte apoptosis in DSS-treated animals, which was substantially prevented by AS-IV (Figure 6E). Furthermore, pro-apoptotic signaling molecules—including phosphorylated JNK (p-JNK), Bax, and cleaved caspase-3—were elevated in DSS-exposed livers, with the co-treatment of AS-IV markedly reducing their expression (Figure 6F). These findings indicate that AS-IV effectively mitigates DSS-induced hepatic injury by reducing apoptosis and lipid accumulation.

### 3.7. AS-IV Diminishes Expression of Oxidative Stress Markers and Suppresses MAPK and NF-κB Signaling in Hepatic Tissue of DSS-Induced Acute Colitis Mice

**Figure 7:**
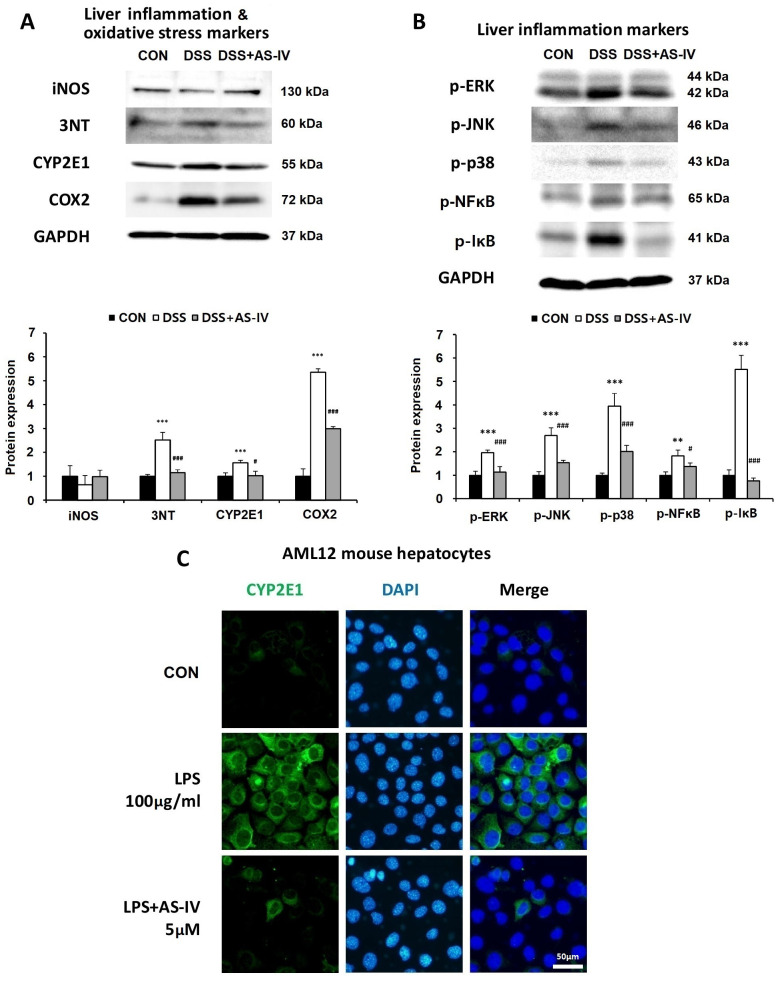
AS-IV attenuates hepatic oxidative/nitrosative stress and inhibits MAPK and NF-κB signaling in DSS-induced colitis. Liver tissue samples were obtained from CON, DSS, and DSS + AS-IV (100 mg/kg) groups. (**A**) Western blot quantification of hepatic inflammatory and oxidative/nitrosative stress markers (iNOS, 3-nitrotyrosine, CYP2E1, COX-2); GAPDH as loading control (*n* = 3 technical replicates from pooled lysates). (**B**) Western blot quantification of phosphorylated MAPK and NF-κB pathway proteins (p-ERK, p-JNK, p-p38, p-NF-κB, p-IκBα) in liver tissue (*n* = 3 technical replicates from pooled lysates). (**C**) Representative immunofluorescence images of CYP2E1 (green) in AML12 hepatocytes treated with CON, LPS (100 μg/mL), or LPS + AS-IV (5 μM); nuclei stained with DAPI (blue) (*n* = 6). Data are presented as means ± SEM. ** *p* < 0.01, *** *p* < 0.005 vs. CON group; # *p* < 0.05, ### *p* < 0.005 vs. DSS or LPS group.

Hepatic levels of 3-nitrotyrosine, COX-2, and CYP2E1 were significantly elevated in DSS-challenged mice, whereas AS-IV treatment markedly reduced these oxidative and nitrosative stress markers (Figure 7A). DSS exposure also substantially elevated the levels of phosphorylated ERK, JNK, and p38, which were reduced by AS-IV administration (Figure 7B). To investigate the anti-inflammatory mechanism of AS-IV via NF-κB signaling, the levels of phosphorylated NF-κB and IκBα were assessed. DSS markedly elevated phosphorylated NF-κB and IκBα levels, whereas AS-IV co-treatment reduced the expression of these phosphorylated proteins (Figure 7B). These findings indicate that AS-IV reduces inflammatory responses and oxidative damage in the liver through inhibition of the NF-κB/MAPK pathways. Additionally, to explore the direct effect of AS-IV on hepatic cells, AML12 hepatocytes were exposed to LPS to induce CYP2E1 expression. Confocal microscopy revealed that AS-IV treatment mitigated LPS-induced CYP2E1 elevation in AML12 cells (Figure 7C).

### 3.8. AS-IV Reduces Expression of Oxidative Stress Markers and Levels of Phosphorylated MAPK Proteins in Neural Tissue of DSS-Induced Acute Colitis Mice and Provides Neuroprotection Against LPS-Mediated Cellular Injury In Vitro

**Figure 8:**
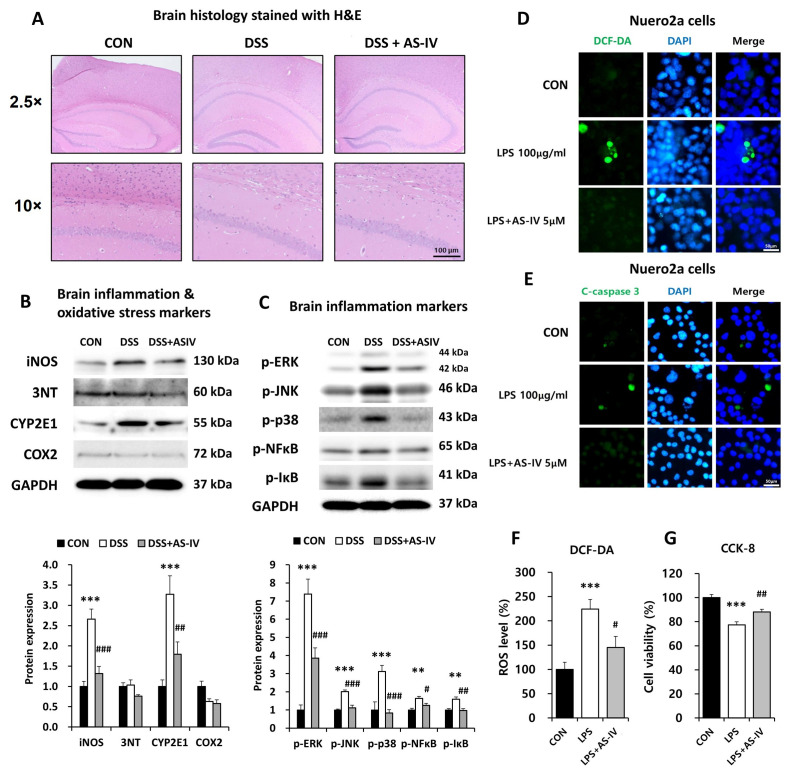
AS-IV attenuates brain oxidative stress and inflammatory signaling in DSS-induced colitis and protects against LPS-induced neuronal injury in vitro. (**A**) Representative H&E-stained brain sections from CON, DSS, and DSS + AS-IV (100 mg/kg) groups (2.5× and 10×; scale bar = 100 μm, *n* = 6). (**B**) Western blot quantification of brain inflammatory and oxidative/nitrosative stress markers (iNOS, 3-nitrotyrosine, CYP2E1, COX-2); GAPDH as loading control (*n* = 3 technical replicates from pooled lysates). (**C**) Western blot quantification of phosphorylated MAPK and NF-κB pathway proteins (p-ERK, p-JNK, p-p38, p-NF-κB, p-IκBα) in brain tissue (*n* = 3 technical replicates from pooled lysates). Representative immunofluorescence images of DCF-DA (**D**) and cleaved caspase-3 (**E**) in Neuro2a cells treated with CON, LPS (100 μg/mL), or LPS + AS-IV (5 μM); nuclei stained with DAPI (blue) (*n* = 6). (**F**) Quantification of intracellular ROS levels through DCF-DA assay. (**G**) Cell viability assessed through CCK-8 assay. Data are presented as means ± SEM. ** *p* < 0.01, *** *p* < 0.005 vs. CON group; # *p* < 0.05, ## *p* < 0.01, ### *p* < 0.005 vs. DSS or LPS group.

To evaluate AS-IV’s potential in preventing neurological dysfunction associated with DSS-induced colitis via the gut–brain axis, oxidative stress and inflammatory markers were assessed in neural tissues using immunoblotting and histological analyses. H&E staining revealed minimal morphological alterations across DSS and DSS plus AS-IV (100 mg/kg) groups (Figure 8A). DSS exposure markedly increased neural iNOS and CYP2E1 levels, indicative of oxidative and nitrosative stress, which were substantially reduced by AS-IV treatment (Figure 8B). The levels of phosphorylated ERK, JNK, and p38 were also elevated in DSS-treated neural homogenates, whereas AS-IV administration reduced the expression of these phosphorylated proteins (Figure 8C). These results suggest that AS-IV mitigates neuronal oxidative/nitrosative stress and inflammatory signaling via NF-κB/MAPK pathway modulation. To further assess AS-IV’s neuroprotective effects against LPS-induced cellular injury, N2a neuroblastoma cells were exposed to LPS with or without AS-IV treatment. Confocal microscopy demonstrated increased ROS production and elevated cleaved caspase-3 levels in LPS-treated cells, both of which were significantly attenuated by AS-IV (Figure 8D,E). These findings were corroborated by ROS quantification and cell viability assays, confirming that AS-IV reduces LPS-induced oxidative stress and preserves neuronal integrity (Figure 8F,G). Overall, these observations indicate that AS-IV protects neural tissue from damage induced by DSS or LPS through the suppression of oxidative stress, apoptosis, and inflammatory signaling.

## 4. Discussion

IBD constitutes a collection of multifaceted gastrointestinal pathologies characterized by dysregulated mucosal immune mechanisms affecting intestinal epithelial tissue [36]. Contemporary pharmacological investigations into potential anti-inflammatory modalities, including experimental and clinical studies, persist in demonstrating the therapeutic efficacy of natural compounds for managing multiple autoimmune disorders, specifically IBD [36]. Moreover, AS-IV displays considerable protective potential against inflammatory mechanisms and oxidative cellular damage via intestinal epithelial dysfunction inhibition [37], proposing its utility as a therapeutic agent within traditional pharmaceutical methodologies. Regarding safety, previous studies have demonstrated that AS-IV exhibits a favorable toxicological profile, with no significant hepatotoxicity or nephrotoxicity observed following oral administration in rodents [1,28]. The doses employed in the present study (10–200 mg/kg) are consistent with those used in prior experimental studies and are considered to be within a safe range based on available preclinical evidence [1,28]. The reduced efficacy observed at 200 mg/kg compared to 100 mg/kg is unlikely to reflect toxicity, as no adverse clinical signs were observed at any dose; however, the precise mechanism underlying this dose–response pattern warrants further investigation.

Although the mechanisms underlying CYP2E1 upregulation within DSS-exposed animals remain incompletely understood, preceding investigations have demonstrated that DSS-mediated intestinal epithelial disruption precipitates heightened bacteria-synthesized alcohol concentrations, particularly originating from *Escherichia coli* populations, potentially promoting endogenous ethanol production. This heightened ethanol concentration may subsequently activate CYP2E1 expression throughout hepatic compartments, intestinal mucosa, renal tissue, and neurological structures, as previously documented in animal model systems receiving fructose administration [38]. Additional mechanistic investigation represents an important avenue for future research endeavors. The interdependency of inflammatory mechanisms and oxidative cellular dysfunction constitutes a fundamental pathophysiological linkage, whereby both elements substantially influence disease progression and the advancement of diverse pathological conditions within inflammatory pathologies [39]. AS-IV treatment demonstrates a documented suppression of inflammatory processes accompanied by substantial anti-inflammatory manifestations [1]. These collective observations recommend that AS-IV exhibits therapeutic promise as a therapeutic intervention for numerous persistent inflammatory conditions and associated complications [1,40,41,42].

The DSS-challenged experimental colitis paradigm is one of the most widely used models for gastrointestinal inflammation research [43]. Clinical symptoms including body weight loss, intestinal mucus output, frequent loose stools, and intestinal hemorrhage constitute established markers for evaluating pathological advancement within DSS-triggered colitis circumstances. Within our experimental colitis scenario, dextran sulfate sodium contact generated substantial gastrointestinal inflammatory manifestations characterized by considerable body mass diminution, recurrent loose stool passage, and hemorrhagic intestinal events, quantifiable via DAI documentation. AS-IV treatment attenuated DSS-induced colitis severity; this reduction in colitis intensity was accompanied by preserved epithelial barrier integrity and reduced inflammatory cell infiltration in both mucosal and submucosal layers. Through mechanistic investigation, we assessed inflammatory cascade modifications inside DSS-triggered IBD specimen populations. Inflammatory phagocytes contribute substantially to inflammatory cascade involvement via pro-inflammatory substance secretion encompassing IL-1β and IL-6, alongside supplementary inflammatory molecules including nitrogen monoxide [44]. Within our experimental scheme, the suppression of TNF-α, IL-1β, and NO production by AS-IV is consistent with its well-documented anti-inflammatory properties reported in previous studies [1,40,41,42]. Consequently, AS-IV manifests characteristics of suppressing inflammatory signal activation and regulating immune mechanism operation throughout the DSS-triggered IBD animal paradigm.

In the present study, AS-IV reduced the levels of phosphorylated IκBα (Figure 4D, Figure 7B and Figure 8C), thereby attenuating NF-κB nuclear translocation and the consequent upregulation of iNOS and COX-2 in DSS-challenged mice. These findings suggest that AS-IV exerts its anti-inflammatory effects at least in part through a reduction in phosphorylated IκBα levels and subsequent suppression of NF-κB activation across colonic, hepatic, and neural tissues. Equivalent information regarding AS-IV’s function as an inflammatory suppressor via NF-κB pathway regulation has been documented across separate investigations [28,45,46,47].

The findings substantiate that lipopolysaccharide drives inflammatory processes and oxidative damage throughout diverse tissue compartments, particularly hepatic and neurological constructs. Epithelial barrier disruption and intestinal epithelial dysfunction are consequences of elevated lipopolysaccharide concentrations and supplementary detrimental microbial metabolites/substances, adding to the IBD process. Moreover, information indicates that alternative pathological mechanisms, primarily neurodegenerative pathologies, undergo initiation via intestinal–hepatic–cerebral connection within IBD sufferers. Throughout our examination, the attenuation of hepatic apoptosis and lipid accumulation by AS-IV is consistent with the reduction in systemic LPS levels, supporting the concept that intestinal barrier restoration limits inflammatory signal propagation to the liver via the gut–liver axis. LPS-mediated TLR4 activation has been shown to upregulate pro-inflammatory mediators and oxidative stress markers in both hepatic and neural tissues [48,49], consistent with the molecular changes observed in the present study. Furthermore, elevated systemic LPS has been reported to compromise the integrity of the blood–brain barrier (BBB), thereby facilitating neuroinflammatory responses through TLR4-mediated signaling and promoting neuronal apoptosis via the upregulation of pro-inflammatory mediators including TNF-α, COX-2, and IL-1β [50,51]. In the present study, DSS-challenged mice exhibited significantly elevated neural expression of iNOS, CYP2E1, COX-2, and phosphorylated MAPK/NF-κB proteins, providing indirect molecular evidence of neuroinflammatory modulation along the gut–brain axis. These findings were further supported by in vitro experiments demonstrating that AS-IV attenuated LPS-induced ROS accumulation, cleaved caspase-3 expression, and cell death in N2a neuroblastoma cells [52,53]. However, it should be noted that direct functional neurological assessments were not performed in this study, and the observed molecular changes should be interpreted as gut–brain axis-associated neuroinflammatory alterations rather than definitive evidence of gut–brain axis modulation. Future investigations incorporating behavioral assessments and BBB permeability assays would be warranted to formally substantiate these findings.

A number of limitations inherent to the present study warrant careful consideration. First, only male mice were used in this study, as female mice exhibit cyclical fluctuations in estrogen levels that may influence inflammatory responses and confound the interpretation of results in the DSS-induced colitis model. However, we fully recognize that this approach limits the generalizability of our findings, given that sex-dependent differences in immune responses and susceptibility to intestinal inflammation have been reported in the literature. Therefore, potential sex-dependent differences in the response to AS-IV treatment cannot be excluded, and future studies incorporating both male and female mice are warranted to determine whether the protective effects of AS-IV are consistent across sexes. Second, although we observed elevated CYP2E1 expression across intestinal, hepatic, and neural tissues, the precise upstream mechanisms driving this increase under DSS-induced colitis conditions remain to be fully elucidated. Third, no environmental enrichment was provided during the experimental period, which may have influenced stress-related parameters. Fourth, the in vitro experiments employed both human (T84) and murine (AML12, N2a) cell lines, which introduces a degree of species heterogeneity. Although each cell line was selected based on its established relevance to the corresponding tissue model, future studies utilizing species-matched cell lines throughout would further enhance the interpretive consistency of the findings. Fifth, although NRF2/KEAP1 signaling is closely associated with oxidative stress regulation and would provide additional mechanistic insight into the antioxidant effects of AS-IV, the potential involvement of NRF2/KEAP1 signaling in the antioxidant effects of AS-IV was not examined in the present study, and future investigations should address this pathway along the gut–liver–brain axis. Sixth, while AS-IV was associated with the suppression of NF-κB and MAPK signaling across colonic, hepatic, and neural tissues, the present study provides correlative rather than causal evidence. Future studies incorporating selective pathway inhibitors or genetic knockdown approaches would be necessary to formally establish the mechanistic causality of these findings. Seventh, while molecular markers of neuroinflammation and oxidative stress were significantly modulated in neural tissues and neuroprotective effects were demonstrated in vitro using N2a cells, direct functional neurological assessments such as behavioral tests and blood–brain barrier permeability assays were not performed in the present study. Future studies incorporating these functional endpoints would be necessary to formally substantiate the gut–brain axis conclusions. Eighth, Western blot analyses were performed on pooled protein lysates prepared from all mice within each group, which precludes the calculation of within-group variance and limits the statistical inference of these data. All other quantitative assays were performed on individual animal samples. Future studies will perform Western blot analyses on individually prepared lysates to enable full statistical evaluation. Ninth, the present study did not include an AS-IV-alone control group (i.e., mice receiving AS-IV without DSS treatment), which limits the ability to fully assess the intrinsic safety and potential physiological effects of AS-IV administration in the absence of colitis. Although prior preclinical studies have reported a favorable safety profile for AS-IV at doses comparable to those used in the present study [1,28], future investigations incorporating an AS-IV-only control group would be warranted to more comprehensively characterize its safety under non-inflammatory conditions. These limitations should be addressed in future investigations.

Grounded in our information, our current investigation reveals that physiologically suitable dosing concentrations of AS-IV display considerable shielding consequences throughout the intestinal–hepatic–cerebral connection throughout DSS-triggered IBD laboratory subjects via inflammatory substance generation prohibition and pro-inflammatory cascade element inhibition accomplished by the suppression of NF-κB and MAPK pathway regulation mechanisms, oxidative/nitrosative damage, epithelial partition dysfunction, and mobile deterioration. Considering our observations, we suggest that AS-IV deserves additional evaluation as a promising therapeutic candidate for managing inflammatory pathologies including colitis.

## 5. Conclusions

Taken together, the results of this investigation reveal that AS-IV exerts broad protective actions against both the intestinal pathology and systemic complications arising from DSS-induced acute colitis. AS-IV significantly alleviated clinical symptoms, preserved colonic architecture, and reduced oxidative stress and inflammatory responses through the inhibition of NF-κB and MAPK signaling pathways.

Importantly, AS-IV conferred multi-organ protection along the gut–liver–brain axis. By reducing systemic endotoxin levels, restoring intestinal microbial balance, and maintaining epithelial barrier integrity, AS-IV limited inflammatory signal propagation beyond the intestine. AS-IV further mitigated hepatic oxidative stress, lipid accumulation, and apoptosis, while suppressing NF-κB/MAPK activation in liver tissue. In neural tissue, AS-IV was associated with the attenuation of oxidative and inflammatory signaling, and demonstrated neuroprotective effects against LPS-induced oxidative stress and apoptotic injury in vitro. However, as direct functional neurological assessments were not conducted, these findings reflect gut–brain axis-associated neuroinflammatory changes and should be further validated in future studies incorporating behavioral and BBB permeability endpoints.

Overall, these findings identify AS-IV as a multi-target antioxidant agent capable of interrupting oxidative stress-driven inflammatory amplification across the gut–liver–brain axis. This study provides mechanistic evidence supporting the therapeutic potential of AS-IV for inflammatory bowel disease and its systemic manifestations, reinforcing the traditional use of *Astragalus membranaceus* Fisch. ex Bunge in gastrointestinal disorders.

## Data Availability

The data presented in this study are available on request from the corresponding author. The data are not publicly available due to privacy and ethical restrictions.

## Supplementary Materials

The following supporting information can be downloaded at: https://www.mdpi.com/article/10.3390/antiox15040474/s1, Table S1: System of scoring to calculate the disease activity index (DAI).

## Abbreviations

The following abbreviations are used in this manuscript:

AJ	adherens junction
ALT	alanine aminotransferase
AS-IV	Astragaloside IV
BBB	blood–brain barrier
CD	Crohn’s disease
DAI	disease activity index
DAPI	4′,6-diamino-2-phenylindole
DCF-DA	2′,7′-dichlorofluorescein diacetate
DMEM	Dulbecco’s modified Eagle’s medium
DSS	dextran sulfate sodium
ELISA	enzyme-linked immunosorbent assay
FBS	fetal bovine serum
H&E	hematoxylin and eosin
IBD	inflammatory bowel disease
IL	interleukin
LPS	lipopolysaccharide
NO	nitric oxide
RIPA	radioimmunoprecipitation assay
ROS	reactive oxygen species
TG	triglyceride
TJ	tight junction
TUNEL	terminal deoxynucleotidyl dUTP nick end labeling
UC	ulcerative colitis
