# Supplementary material for: Astragaloside IV from Astragalus membranaceus Fisch. ex Bunge Mitigates DSS-Induced Colitis via Anti-Inflammatory and Antioxidant Modulation of the Gut–Liver–Brain Axis
Source: Antioxidants (Basel). 2026 Apr 10;15(4):474. doi: 10.3390/antiox15040474 (PMC13114091; doi:10.3390/antiox15040474)
Supplement: Supplementary file 1 [file antioxidants-15-00474-s001.zip › antioxidants-4184687-supplementary.pdf]

Supplementary Materials.

Table S1. System of scoring to calculate the disease activity index (DAI). The DAI value is obtained by averaging the scores for weight loss, stool consistency, and presence of blood in feces.

Score	Weight loss	Stool consistency	Visible blood feces
0	None	Normal	None
1	1~5%		
2	6~10%	Loose	Slight bleeding
3	11~20%		
4	<20%	Diarrhea	Gross bleeding
